# The Risk of Recurrence of Subacute Thyroiditis Is HLA-Dependent

**DOI:** 10.3390/ijms20051089

**Published:** 2019-03-03

**Authors:** Magdalena Stasiak, Bogusław Tymoniuk, Bartłomiej Stasiak, Andrzej Lewiński

**Affiliations:** 1 Department of Endocrinology and Metabolic Diseases, Polish Mother’s Memorial Hospital - Research Institute, 93-338 Lodz, Poland; mstasiak33@gmail.com; 2 Department of Immunology, Rheumatology and Allergy, Medical University of Lodz, 92-213 Lodz, Poland; boguslaw.tymoniuk@umed.lodz.pl; 3 Institute of Information Technology, Lodz University of Technology, 90-924 Lodz, Poland; bartlomiej.stasiak@p.lodz.pl; 4 Department of Endocrinology and Metabolic Diseases, Medical University of Lodz, 93-338 Lodz, Poland

**Keywords:** subacute thyroiditis, recurrence, HLA, risk factor, steroid dose

## Abstract

The frequency of recurrence of subacute thyroiditis (SAT) is rather high, reaching 20–30%. The reason for SAT relapse is still unknown. Recently, we have demonstrated the association between SAT and the presence of HLA-B*18:01, DRB1*01, and C*04:01, apart from the previously known HLA-B*35. The aim of the present study was to evaluate the correlation between SAT-associated HLA haplotypes and the risk of SAT recurrence. HLA-A, -B, -C, -DQB1 and -DRB1 were genotyped using a next-generation sequencing method in 49 SAT patients. The patients were divided into the following HLA groups: 1. HLA-B*35 and/or HLA-C*04, but without any other of the analyzed antigens; 2. HLA-DRB1*01, regardless of the co-presence of HLA-B*35 or -C*04:01, but without HLA-B*18:01; 3. HLA-B18 only, without any other antigen; 4. HLA-B*18:01 plus -B*35, regardless of the presence of any other analyzed antigens. The recurrence rate was compared between the groups. The recurrence rate was significantly increased in patients with HLA-B*18:01 plus HLA-B*35. In conclusion, the risk of SAT recurrence was HLA-dependent and the determining factor was the co-presence of HLA-B*18:01 and -B*35. In such high-risk patients, the steroid treatment regimen should be intensified with a slower dose reduction.

## 1. Introduction

Subacute thyroiditis (SAT, also called granulomatous thyroiditis or de Quervain’s thyroiditis) is a rare inflammatory thyroid disease, triggered probably by a preceding viral infection (occurring approximately 2–6 weeks earlier). The prevalence of SAT is four to seven times higher in women than in men [1,2] and most patients are middle-aged [2].

The most common complaint of patients with SAT is anterior neck pain, typically radiating ipsilaterally up to the jaw and ear and to the upper mediastinum. Fever occurs in the majority of cases, reaching frequently over 39 °C, and rising especially at night. All the initial symptoms may be accompanied by muscle pain, fatigue and malaise. Laboratory markers of mild to moderate hyperthyroidism are often found, but the levels of anti-thyroid antibodies are normal in over 70% of patients [2,3,4,5]. In some individuals, symptoms of thyrotoxicosis are present, usually of low to moderate severity [3,4]. In rare cases, they dominate the clinical presentation, with a medical history of weight loss, tremor and palpitations. Thyrotoxicosis (usually lasting for 2 to 8 weeks) is a result of the destruction of thyroid follicles and the release of thyroid hormones. A subsequent hypothyroid phase does not occur frequently, and a persistent one is extremely rare in the SAT course [3,4]. In laboratory findings, a high erythrocyte sedimentation rate (ESR), sometimes reaching even three-digit values, is a characteristic feature of SAT. C reactive protein (CRP) is also usually elevated. The ultrasound (US) features of SAT include the presence of hypoechoic and heterogeneous areas with blurred margins, poorly vascularized on color Doppler ultrasound [6,7]. Firm thyroid nodules in SAT can grow rapidly, initially suggesting thyroid malignancy. The acute symptoms of the disease frequently last for weeks or even for months, and significantly hinder patients’ normal functioning. In some cases, the SAT course is of mild severity and the disease subsides spontaneously, with ending of pain and fever and normalization of the hormonal parameters (approximately after 8–16 weeks). However, in many patients, the resolution of symptoms can be achieved only by a medical treatment. First-line therapy is the application of nonsteroidal anti-inflammatory drugs (NSAIDs), while glucocorticoids are used in cases of NSAID ineffectiveness. Prednisone is the most commonly used steroid, starting from high doses of even over 40 mg a day. In some patients, SAT relapses once or even several times.

Even in patients treated properly, the rate of SAT recurrence is rather high, varying significantly between studied groups, ranging from 1.6% [5] to over 20% [8]. The observed discrepancies between different studies seem to be dependent on the studied population (Caucasian vs. Asian). Recurrences of SAT may occur soon after the completion of treatment, but they also happen even after many years from the first episode [9,10]. The occurrence of one relapse increases the risk of the next one. The recurrence of symptoms often occurs when the dose of prednisone is tapered to 5–10 mg. The recurrence rate during treatment with prednisolone was reported to range from 2.2% to 35% [11]. In such patients, the tapering regimen should be very slow, and prolonged steroid treatment is required [12].

Long-lasting treatment with glucocorticoids is associated with several adverse effects, such as acne, skin thinning, bruising, mood changes, hyperhidrosis, weight gain, edema, cushingoid body habitus, depression, hypertension, hyperglycemia and osteoporosis. Thus, the determination of the risk groups for recurrent SAT would allow adequate treatment to prevent early recurrence. Additionally, the awareness of the risk of a long-time relapse would enable early diagnosis and treatment.

The reason for SAT relapse is still unknown. Some authors postulate that a too short period of steroid therapy is the causative factor [11]. Shortening of the time of steroid therapy may result in the recurrence of SAT symptoms due to treatment withdrawal before the complete SAT resolution. However, a short-time steroid regimen may be associated with a higher risk of SAT recurrence, even in patients with complete remission after therapy of the first episode of the disease [11]. Additionally, the SAT recurrence occurs also in several percent of patients treated with steroids according to the prolonged regimen (i.e., starting from 40 mg of prednisone and tapering the dose for 5 mg every week) [2,11]. In some patients, even steroid dependence occurs, and any attempt to discontinue treatment results in SAT relapse. Thus, other factors seem to be more crucial for the risk of recurrence than the period of steroid treatment. Nevertheless, the longer time of steroid administration seems to be protective.

Since 1977, the susceptibility to SAT has been known to be HLA-B*35 related in approximately 70% of patients [13,14,15]. Recently, we have demonstrated the association between SAT and the presence of HLA-B*18:01 and DRB1*01, as well as HLA-C*04:01, with the latter being in linkage disequilibrium with HLA-B*35 [16]. Haplotypes of HLA-B*18:01 and DRB1*01 are HLA-B*35-independent SAT risk factors [16]. These new three antigens, together with previously known HLA-B*35, allow confirmation of the genetic basis in almost all patients with SAT. Using the old serologic method of HLA typing, Yamamoto at al. [9] postulated the correlation between SAT recurrence and HLA-A26, but those findings have never been confirmed. Moreover, no dependence of HLA-A*26 (current nomenclature) and SAT has ever been demonstrated.

The association between the type of the known genetic SAT background and the risk of the disease recurrence has never been analyzed. The primary goal of the study was to evaluate the potential correlation between the presence of SAT-associated HLA haplotypes, i.e., HLA-B*18:01, DRB1*01, B*35 and C*04:01, and the risk of recurrence of the disease. The secondary aim of the study was to compare the clinical and laboratory features of patients with (recurrence group, or RG) and without (non-recurrence group, or NRG) SAT recurrence, to find out the potential clinical prognostic factors of SAT relapse.

## 2. Results

Nine patients (14%) reported SAT recurrence (at least once). No differences in patient age between the NRG and RG were observed. The mean age of patients in the NRG was 42.7 years and in the RG – 44.4 years (*p* = 0.675). The male to female ratios were 1:4.7 in NRG, and 1:8 in RG (*p* = 1.000).

**Figure 1:**
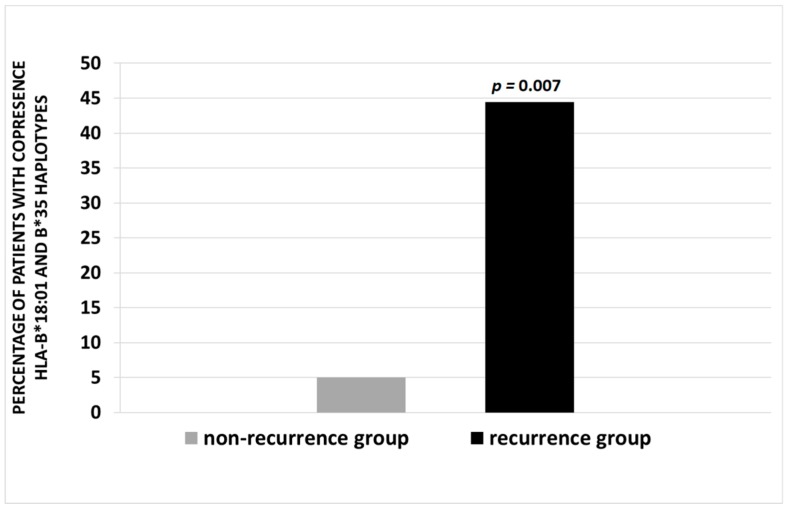
Percentage of patients with co-presence of HLA-B*18:01 and B*35 haplotypes in non-recurrence (NRG) and recurrence (RG) groups. A *p* value of <0.05 is considered statistically significant.

The recurrence rate was significantly increased in patients with HLA-B*18:01 plus HLA-B*35 haplotypes. The frequency of SAT recurrence in this group was 44.4%, vs. 5% in patients without the co-presence of these two haplotypes (*p* = 0.007) (Figure 1).

The incidences of SAT relapses in all other analyzed HLA groups were not significantly increased (Table 1). In three of NRG patients, none of the analyzed sets of SAT high-risk HLA antigens were found.

No significant differences were observed between the RG and NRG, concerning the presence of such clinical symptoms as neck/ear pain (*p* = 0.569), fever (*p* = 1.000) and preceding infection (*p* = 0.702) (Table 2). 

Laboratory markers of thyrotoxicosis were significantly more severe in the NRG than in the RG. The levels of thyroid stimulating hormone (TSH), free triiodothyronine (FT3), free thyroxine (FT4) and anti-thyroid peroxidise antibodies (aTPO) were significantly different between the NRG and RG. The laboratory results for the NRG and RG are presented in Table 3.

No other significant differences were found in the analyzed biochemical parameters, such as thyroglobulin antibodies (aTg), thyrotropin receptor antibodies (TRAb), ESR, CRP, white blood count (WBC), 25-hydroxy vitamin D (Table 3).

## 3. Discussion

Subacute thyroiditis is a rare disease but its prevalence is increasing. In Caucasian patients, a recurrence rate of several percent is usually reported [8,17], and indeed, in our cohort, nine patients (14%) reported SAT recurrence (at least once).

Recently, the correlation between the SAT occurrence and the presence of HLA-B*18:01 and DRB1*01, as well as HLA-C*04:01 has been demonstrated by our research group, with the latter one being in linkage disequilibrium with a well-known SAT risk haplotype HLA-B*35 [16]. These new three antigens, together with the known HLA-B*35, allow confirmation of the genetic basis in almost all patients with SAT. The haplotypes HLA-B*18:01, -DRB1*01 and HLA-B*35 are all independent SAT risk factors [16]. The risk of SAT recurrence has never been analyzed in these groups of SAT risk haplotypes. The only association suggested so far was based on serological HLA typing. Using this method, Yamamoto at al. [9] postulated the correlation between SAT recurrence and HLA-A26. No other author confirmed such correlation, not even a dependence of HLA-A*26 (current nomenclature) and SAT. In our cohort, the HLA-B*26 haplotype was present in one patient only, and this patient belonged to the NRG. Thus, no correlation has been observed.

However, our present study has demonstrated for the first time that the risk of SAT recurrence is indeed HLA-dependent, and the high-risk group includes patients with co-occurrence of HLA-B*18:01 and -B*35. It seems that the presence of HLA B18:01 significantly changes the course of the SAT. We have recently demonstrated that the ultrasound SAT pattern in patients with HLA-B*18:01 is different from the typical image for the disease [18]. The risk of recurrence was significantly influenced by the presence of HLA-B*18:01, but only with the concurrent presence of HLA-B*35. The HLA-B*35 haplotype alone did not increase the SAT recurrence rate. It is difficult now to unequivocally explain this phenomenon. It can be speculated, that the presence of HLA-B*18:01 changes the course of SAT, but the co-occurrence of HLA-B*35 is required so that the combined effect of the presence of the two independent key SAT risk haplotypes significantly increases the risk of recurrence.

Demonstration that the co-occurrence of HLA-B*18:01 and -B*35 carries the risk of SAT recurrence should be confirmed in further studies, which have already been initiated in our center. Definitive confirmation of our present findings will allow to introduce them into clinical practice by adjusting the steroid treatment regimen in patients with high risk of relapse and recommending very slow dose tapering (e.g., a reduction of 5 mg every 10–14 days). Currently, HLA typing is quite widely available, and the possibility of limiting the scope of the analyzed HLA antigens to the specific high-risk ones makes it also affordable.

Data on the clinical and laboratory differences between the NRG and RG are scarce. In our study, no differences were observed between the RG and NRG with regard to the presence of clinical symptoms, including neck/ear pain, fever or the medical history of a preceding viral infection. However, biochemical thyrotoxicosis was significantly more pronounced in the NRG than in the RG, with considerably lower TSH and higher FT4 and FT3 levels in the NRG. This observation is surprising, as one would expect the relapse to occur in patients with a more intense inflammatory process. Meanwhile, it seems that increased thyrotoxicosis, resulting from the damage of thyroid follicles, is a predictive factor of a non-relapse SAT course. The degree of thyroid tissue damage seems to play a key role in the risk of recurrence. This hypothesis seems to be confirmed by the fact that in our present study, an elevated aTPO concentration was a very strong factor protecting against relapse. The aTPO levels were within normal range in all RG patients, while they were increased in as much as 18.9% of NRG patients. While an elevated aTg level may be transitory in SAT, due to increased antigen presentation in the acute phase of the disease [19], the presence of aTPO is often persistent and associated with the development of autoimmune thyroid disease (AITD) [2]. The predisposition to AITD is genetically determined and the correlation with several HLA haplotypes was widely postulated [20,21]. The most well-known AITD-related antigen is HLA-DR*3, which belongs to major histocompatibility complex (MHC) class II. On the contrary, both SAT recurrence risk antigens, HLA-B*18:01 and -B*35, belong to MHC class I. Perhaps the presence of HLA antigens typical for AITD protect against the recurrent course of SAT. Additionally, different subpopulations of immune cells can be involved in the inflammatory process in SAT with and without elevated aTPO. Such subpopulations can be HLA-dependent. Further research is necessary to confirm these hypotheses.

No other significant differences were found in the analyzed biochemical parameters, such as aTg, TRAb, ESR, CRP, WBC. Similarly, Mizukoshi et al. [8] found no differences between recurrent and non-recurrent groups in ESR, CRP and WBC levels. On the contrary to our results, these authors observed also no differences in FT3 and FT4 levels between the analyzed groups. However, it is difficult to compare their results to ours, as their groups were smaller and more importantly, no data concerning the levels of thyroid antibodies were provided [8].

Due to the proven pro-inflammatory effect of vitamin D deficiency [22,23], the comparison of serum 25-hydroxy vitamin D levels in the NRG and RG was performed in our study, to find out whether the vitamin D deficiency was a risk factor of SAT recurrence. No differences between the NRG and RG were observed, so neither the vitamin D deficiency nor its severity increases the risk of SAT recurrence. This observation is especially useful in the case of rapid relapses occurring during the reduction of steroid doses or soon after treatment completion. The administration of vitamin D at the first SAT incident does not seem to reduce the risk of rapid recurrence in these patients.

In conclusion, our results provide for the first time evidence that the risk of SAT recurrence depends on HLA haplotype and the determining factor is the co-presence of HLA-B*18:01 and -B*35. In such high-risk patients, the steroid treatment regimen should be intensified with higher initial doses and slower dose reduction. Moreover, more severe biochemical thyrotoxicosis and the presence of elevated aTPO at the first SAT episode may be considered protective factors in regard to SAT recurrence.

## 4. Materials and Methods

The HLA typing was performed in 49 consecutive patients who were diagnosed with SAT between 2003 and 2018 in the Department of Endocrinology and Metabolic Diseases, Polish Mother’s Memorial Hospital—Research Institute, Lodz, Poland. HLA-A, -B, -C, -DQB1 and -DRB1 were genotyped using a next-generation sequencing method on the Illumina platform (Illumina, San Diego, CA, USA). The sequencing-based HLA typing of the HLA genes A, B, C, DQB1 and DRB1, was carried out in 96-well format within a semi-automated workflow by using MiaFora Flex5 typing kits (Immucor, Warren, NJ, USA). The long-range PCR amplification of five HLA loci was performed on DNA extracted from blood samples.

For every patient, the clinical SAT signs and symptoms and laboratory results were established based on the clinical examination and medical history. The recurrence episodes were confirmed on the basis of medical history. After treatment completion, each patient was informed about the need to report symptoms of relapse. Additionally, all patients were monitored for relapse by periodic telephone contact.

The diagnosis of SAT was based on the diagnostic criteria recently proposed by our team [2]. These criteria were as follows: Elevation of ESR (or at least CRP) plus hypoechoic area/areas with a blurred margin and decreased vascularization in US, plus cytological confirmation of SAT (or at least cytological exclusion of malignancy), plus at least one of the following: Hard thyroid swelling and/or pain and tenderness of the thyroid gland/lobe, and/or elevation of serum FT4 and suppression of TSH, and/or decreased RAIU.

Serum levels of TSH, FT3, FT4, aTg, aTPO, TRAb were measured by electrochemiluminescence immunoassay (ECLIA), Cobas e601 analyzer (Roche Diagnostics, Indianapolis, IN, USA), the ESR was determined by Ves-Matic Cube 30 (Diesse, Monteriggioni, Italy), the CRP was determined by VITROS^®^ 4600 Chemistry System (Ortho Clinical Diagnostics, Raritan, NJ, USA), the WBC was determined by flow cytometry, XE-2100 analyzer (Sysmex, Kobe, Japan) and 25-hydroxy vitamin D levels were determined by Liaison XL (DiaSorin S.p.A., Saluggia, Italy). Ultrasound examination was performed in every patient, using a 7-14 MHz linear transducer (Toshiba Aplio XG; Toshiba, Japan). A fine needle aspiration biopsy (FNAB) was performed in all SAT patients using a 23-gauge needle. The smears were cytologically evaluated, and the presence of multinucleated giant cells, together with mononucleated macrophages and follicular epithelial cells against acute and chronic inflammatory dirty background was considered as a result typical for SAT.

All the patients were divided into the following groups according to the HLA haplotype: 1. HLA-B*35 and/or HLA-C*04, without any other of the analyzed antigens; 2. HLA-DRB1*01, regardless of the co-presence of HLA-B*35 or -C*04:01, but without HLA-B*18:01; 3. HLA-B18 only, without any other analyzed antigen; 4. HLA-B*18:01 plus -B*35, regardless of the presence of any other analyzed antigens. The reasons for such group division were as follows: HLA-B*18:01, -DRB1*01 and HLA-B*35 are all independent SAT risk factors. However, HLA-C*04:01 is in linkage disequilibrium with HLA-B*35 in Caucasian populations, and therefore, these two antigens had to be always analyzed together in the same group (Group 1); HLA-DRB1*01 alone, without any other analyzed antigen, was present in one patient only. In most cases, it occurred together with HLA-B*35 or -C*04:01. Thus, this one patient was analyzed in the same Group 2 together with patients with co-existence of HLA-DRB1*01 and HLA-B*35 or -C*04:01, but without another independent risk factor, HLA-B*18:01; HLA-B*18:01 was present either alone or with co-existing HLA-B*35 (± HLA-C*04:01 due to the linkage disequilibrium) with clearly visible differences in the risk of recurrence between these two groups. Thus, patients with HLA-B*18:01 alone were included in Group 3 and patients with co-occurrence of HLA-B*35 ± HLA-C*04:01 were analyzed as Group 4. The incidence of SAT recurrence was analyzed in each group individually and Fisher’s exact test was used to assess statistical significance.

Additionally, all the patients were divided into the RG and NRG, and clinical features (the presence of neck/ear pain, fever, preceding infection) and laboratory findings (levels of TSH, FT3, FT4, ESR, CRP, WBC, 25-hydroxy vitamin D) were compared between these two groups. Descriptive statistics of the collected data included the mean and standard deviation. For comparisons between the groups, we used a Student’s *t*-test for normally distributed variables (i.e., ESR, aTPO and 25-hydroxy vitamin D), the Mann-Whitney U test for the other real-valued data and Fisher’s exact test for categorical variables. The normality of data distributions was assessed by a Shapiro–Wilk test, and in all the tests, a value of *p* < 0.05 was considered significant. The statistical analysis was done with SciPy statistical software tools (scipy.stats/NumPy libraries for Python programming language).

All subjects gave their written informed consent for inclusion before they participated in the study. The study was conducted in accordance with the Declaration of Helsinki, and the protocol was approved by the Ethics Committee of the Polish Mother’s Memorial Hospital—Research Institute, Lodz, Poland (Project identification code—22/2016, approved 9 February 2016).

## Figures and Tables

**Table 1:** Distribution of the analyzed haplotypes in the non-recurrence (NRG) and recurrence (RG) groups.

HLA Haplotypes	NRG (40)	RG (9)	*p* Value
B*35 ± C*04:01	55%	33.3%	0.289
DRB1*01 ± B*35 ± C*04:01	22.5%	11.1%	0.663
B*18:01	10%	11.1%	1.000
B*18:01 + B*35 ± C*04:01	5%	44.4%	**0.007**

A *p* value of <0.05 is considered statistically significant. *P*-value <0.05 is marked in bold. Abbreviations: NRG, non-recurrence group; RG, recurrence group.

**Table 2:** Comparison of the clinical features in the non-recurrence (NRG) and recurrence (RG) groups.

Parameter	NRG (40)	RG (9)	*p* Value
Neck/ear pain	87.5%	100%	0.569
fever	72.5%	77.8%	1.000
preceding infection	32.5%	22.2%	0.702

A *p* value of <0.05 is considered statistically significant. Abbreviations: NRG, non-recurrence group; RG, recurrence group.

**Table 3:** Comparison of the laboratory results in the non-recurrence and recurrence groups.

Parameter (No. of Patients in NRG vs. RG)	Non- Recurrence Group (NRG)		Recurrence Group (RG)		*p* Value	Reference Range and Unit
	Mean ± SD	Abnormal (%)*	Mean ± SD	Abnormal (%)*		
TSH (40 vs. 9)	0.101 ± 0.232	89.8	0.646 ± 0.773	44.4	**0.015**	0.27–4.2 mIU/L
FT4 (39 vs. 9)	3.029 ± 1.604	84.6	1.79 ± 0.628	44.4	**0.006**	0.93–1.7 ng/dL
FT3 (39 vs. 9)	7.237 ± 3.88	69.2	4.332 ± 1.189	44.4	**0.025**	2.6–4.4 pg/mL
aTPO (37 vs. 8)	36.54 ± 63.3	18.9	12.773 ± 3.349	0.0	**0.029**	<34 IU/mL
aTg (36 vs. 8)	140.5 ± 203	27.8	96.9 ± 133	25	0.394	<115 IU/mL
TRAb (32 vs. 7)	1.84 ± 6.99	6.25	1.207 ± 1.851	14.3	0.726	<1.75 IU/mL
ESR (38 vs. 9)	66.6 ± 20	100	59.4 ± 21.5	100	0.381	0-12 mm/h
CRP (36 vs. 9)	9.85 ± 12.3	100	5.08 ± 4.66	100	0.156	<1 mg/dL
WBC (39 vs. 9)	8.938 ± 3.105	25.6	9.894 ± 3.599	44.4	0.322	4.0–10.0 × 10^3^/µL
25-hydroxy vitamin D (27 vs. 9)	22.8 ± 8.356	74	23.9 ± 7.873	66.7	0.736	30-50 ng/mL

* Below the reference range in the case of TSH and 25-hydroxy vitamin D, above the reference range in the cases of all other parameters. A *p*-value of <0.05 is considered statistically significant. *P*-values <0.05 are marked in bold. Data are presented as: mean and standard deviation (SD). Abbreviations: aTg, anti-thyroglobulin antibodies; aTPO, anti-thyroid peroxidase antibodies; CRP, C reactive protein; ESR, erythrocyte sedimentation rate; FT3, free triiodothyronine; FT4, free thyroxine; TRAb, TSH receptor antibodies; TSH, thyrotropin; WBC, white blood cells.

## Abbreviations

AITD	autoimmune thyroid disease
aTg	thyroglobulin antibodies
aTPO	thyroid peroxidase antibodies
CRP	C reactive protein
ECLIA	electrochemiluminescence immunoassay
ESR	erythrocyte sedimentation rate
FNAB	fine needle aspiration biopsy
FT3	free triiodothyronine
FT4	free thyroxine
HLA	human leukocyte antigens
MHC	major histocompatibility complex
NRG	non-recurrence group
RG	recurrence group
SAT	subacute thyroiditis
TRAb	thyrotropin receptor antibodies
TSH	thyrotropin
US	ultrasound
WBC	white blood count
